# Retraction: LncRNA DLX6-AS1 Promotes Malignant Phenotype and Lymph Node Metastasis in Prostate Cancer by Inducing LARGE Methylation

**DOI:** 10.3389/fonc.2021.840152

**Published:** 2021-12-30

**Authors:** Frontiers Editorial Office

The journal and Chief Editors retract the 06 August 2020 article cited above.

Following publication, concerns were raised regarding the validity of the data in the article. The authors failed to provide the raw data or a satisfactory explanation during the investigation, which was conducted in accordance with Frontiers’ policies. Given the concerns, and the lack of raw data, the editors no longer have confidence in the findings presented in the article.

This retraction was approved by the Chief Editors of Frontiers in Oncology and the Chief Executive Editor of Frontiers. The authors have not responded to any correspondence regarding the retraction.

